# Structural insights into the effects of charge-reversal substitutions at the surface of horseradish peroxidase

**Published:** 2016-09

**Authors:** Leila Navapour, Navid Mogharrab

**Affiliations:** 1 Biophysics and Computational Biology Laboratory, Department of Biology, College of Sciences, Shiraz University, Shiraz, Iran; 2 Institute of Biotechnology, Shiraz University, Shiraz, Iran

**Keywords:** Horseradish peroxidase, Charge-reversal substitutions, Molecular dynamics simulation, Stability

## Abstract

Horseradish peroxidase (HRP), has gained significant interests in biotechnology, especially in biosensor field and diagnostic test kits. Hence, its solvent-exposed lysine residues 174, 232, and 241 have been frequently modified with the aim of improving its stability and catalytic efficiency. In this computational study, we investigated the effects of Lys-to-Glu substitutions on HRP structure to model charge-reversal manipulations at the enzyme surface. Simulation results implied that upon these substitutions, the number of stable hydrogen bonds and α-helical content of HRP are increased and the proximal Ca2+ binding pocket becomes more integrated. The results revealed that although Glu174-heme hydrogen bond is lost after mutation, formation of a new hydrogen bonding network contributes to the stability of heme-protein linkage. Together, it may be concluded that these substitutions enhance the stability of the protein moiety as well as the heme-protein non-covalent interactions. In the enzyme active site, we observed increased accessibility of peroxide binding site and heme prosthetic group to the peroxide and aromatic substrates, respectively. Results also demonstrated that the bottleneck entry of the peroxide-binding site has become wider and more flexible upon substitutions. Moreover, the hydrophobic patch functioning as a binding site or trap for reducing aromatic substrates is more extended in mutated enzyme. These observations suggest that the reactivity of the enzyme to its substrates has increased. Together, the results of this simulation study could provide possible structural clues to explain those experimental observations in which the protein stability achieved upon manipulation of charge distribution on protein surface.

## INTRODUCTION

Peroxidases have found diverse applications in various industrial and biotechnological fields [1,2]. Among them, horseradish peroxidase C, has gained significant interests in biotechnology, especially in biosensor field and diagnostic test kits [2-6]. Hence, a large body of research involving chemical and genetic modifications of HRP has been accomplished to improve its conformational and operational characteristics. Several successful efforts have been made through the targeting of surface accessible lysine residues [7-16]. No stability gain was observed from modification of other residues such as histidine, arginine, tyrosine, aspartic and glutamic acids [17, 18]. Several reports indicate that modification of lysine residues in a number of other enzymes such as α-amylase, aminotransferase, trypsin, papain and stem bromelain also increases the enzyme stability [19-23]. Although such studies are numerous, but the mechanism underlying the observed improvements in HRP stability upon lysine modification has remained undetermined.

HRP C is a heme and calcium containing glycoprotein with six lysine residues, among them lysine residues 174, 232 and 241 are located on the protein surface [24, 25]. It has been reported that thermostability of HRP increases by chemical modification of these three accessible lysines, but the stabilizing effect is lost after complete modification of all six lysine residues [7]. Most of the stabilized derivatives of HRP reported to date have involved chemical or genetic manipulations that neutralize or reverse the positive charges on the accessible lysine residues. Accordingly, these modifications can be classified into three distinct groups: The first and second are those which alter lysines to neutralized aromatic or aliphatic hydrophobic residues. The third group, are charge-reversing modifications. In a previous study, we modeled a case from the first group by mutating the accessible lysine residues to phenylalanine. Modeling and simulation results clarified some structural clues relating to stability enhancement including decreased flexibility of the protein backbone as well as heme prosthetic group, increased number of stable hydrogen bonds, improved heme-protein interactions and more integrated proximal Ca2+ binding pocket. However the stability gain has been achieved at the expense of reduced activity [26]. In the present study, we try to investigate the effects of the second group of modifications on the stability and activity of HRP. Hence, lysine residues of 174, 232 and 241 were computationally replaced by glutamic acid residues to mimic the charge-reversing chemical or genetic modifications.

## MATERIALS AND METHODS

The starting atomic coordinate of native HRP (n-HRP) was obtained from Protein Data Bank (PDB ID: 1ATJ) [27]. To generate the initial structure of the modified protein (g-HRP), the lysine residues 174, 232 and 241 of native HRP were replaced by glutamic acid residues. All molecular dynamics simulations were performed using the GROMACS simulation package version 5.0.5 [28] and GROMOS96 force field [29]. Each protein was centered in a cubic box and immersed in SPC water molecules so that the shortest distance between the protein and the box boundaries was 1.3 nm and periodic boundary conditions were applied. In order to neutralize the systems, adequate number of water molecules was replaced with Cl– or Na+ ions. Each solvated and neutralized system was energy-minimized using the steepest descents algorithm until the maximum force was smaller than 500 kJ mol-1 nm-1. After energy minimization, two separate position-restrained MD simulations were sequentially carried out to equilibrate the system. First, to adjust the system temperature, an NVT MD simulation was performed for 200 picoseconds (ps) at 300 K by imposing thermal energy in a constant volume condition using the velocity rescale algorithm (modified Berendsen thermostat) with τT= 0.1 ps [30,31]. After arrival at the correct temperature, the resulting atom velocities and coordinates were used to start an NPT MD simulation at 300 K and 1 bar for 200 ps by the Parrinello-Rahman algorithm with τP = 2.0 ps during which density of the system was stabilized at around 1000 kg m-3 [32]. Finally, the production MD period of 100 nanoseconds (ns) at constant pressure (1 bar) and temperature (300 K) without position restraints was performed on native and g-HRP. Bond lengths were constrained using LINCS algorithm [33]. Lennard-Jones (LJ) and short-range electrostatic interactions were calculated with 1.4-nm cutoffs and a particle mesh Ewald algorithm was used for the long range electrostatic interactions [34]. A time step of 2 fs was used for the integration of equation of motion. The Eris web server was used to predict possible changes in the structural stability of HRP after substitutions [35].

## RESULTS

**Figure 1:**
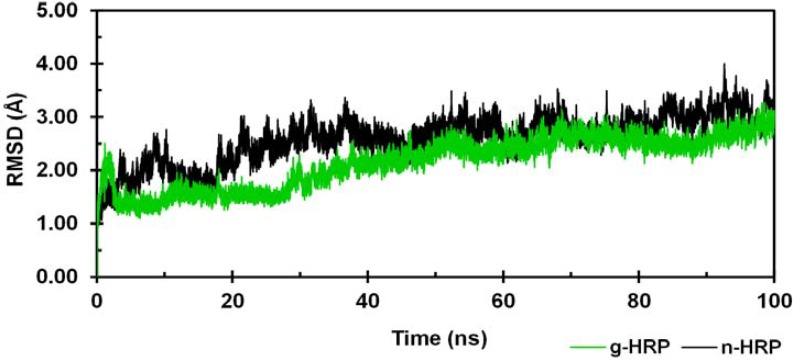
Time dependence of the backbone RMSD with respect to the starting structure during the entire course of 100 ns MD simulations. n-HRP (black) and g-HRP (green) are reported

**Figure 2:**
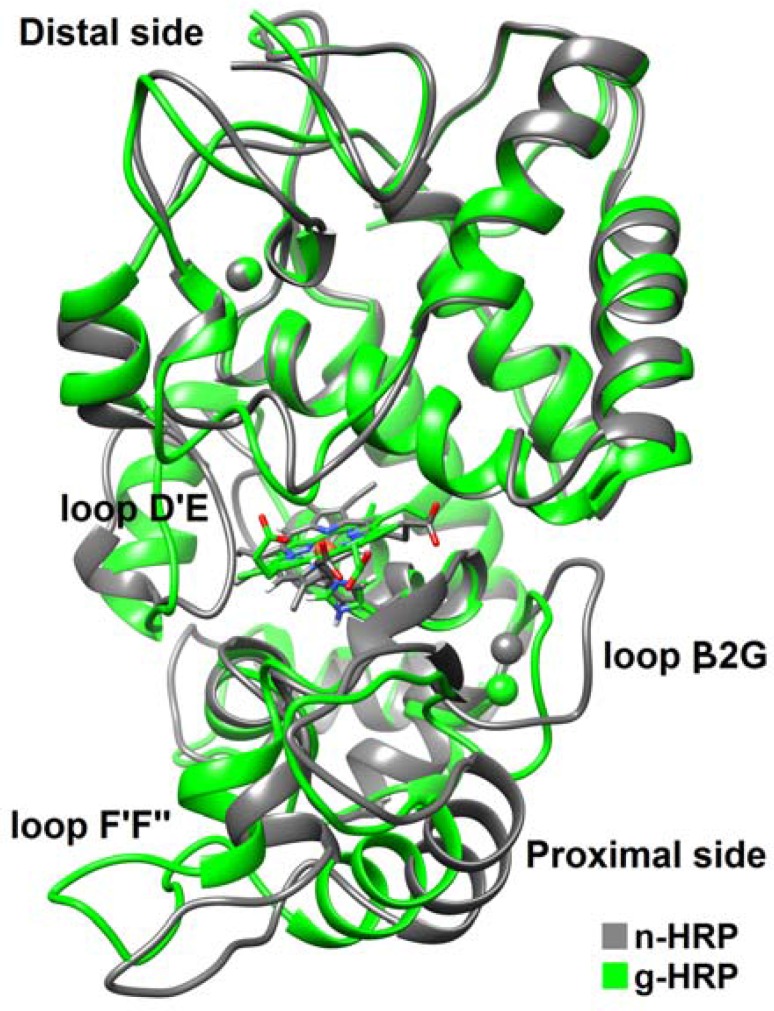
Superimposition of the average structures of n-HRP (gray) and g-HRP (green) obtained by averaging from the trajectories of the analyzed time frames. The positions of the loops D'E, F'F", and β2G are indicated

Using the known x-ray crystallographic structure of HRP C (PDB ID: 1ATJ), two 3D molecular models of HRP C, n-HRP and g-HRP, were constructed differing in the residues 174, 232 and 241. The overall stability and structural relaxation of the enzymes were monitored by computing time evolution of the root mean square deviation (RMSD) of the backbone atoms along the simulations. The backbone RMSDs of n-HRP and g-HRP with respect to their starting structure were calculated to be 2.59 ± 0.45 and 2.16 ± 0.48 Å, respectively (Fig. 1). According to the crystallographic structure of native HRP (PDB ID: 1ATJ), thirteen α-helices dominate the structure: 14-28 (A), 32- 44 (B), 77-90 (C), 97-111 (D), 131-137 (D'), 145-153 (E ), 160-171 (F), 181-185 (F'), 199-208 (F"), 232-238 (G), 245-252 (H), 260-267 (I), and 270-284 (J). Two short antiparallel β-strands, 174-176 (β1) and 218-220 (β2), flank the large plant peroxidase insert between helices F and G. RMSD analysis of the protein backbone indicates that both proteins have experienced sensible structural changes during the early nanoseconds of the simulations. The large increase in the backbone RMSD value during the first 2 ns of g-HRP simulation is mainly due to structural rearrangement of the loop F'F". As seen in Fig. 1, the backbone RMSD of both models with respect to their starting structures appears to be less fluctuating after 50 ns of simulation. Hence, our analyses were focused on those trajectories obtained from the second half of each simulation. Ribbon representations of the average structures obtained from the trajectories of the analyzed time window are shown in Fig. 2. HRP is composed of two spatially distinct domains, namely proximal and distal. There is one calcium ion per each domain and the heme prosthetic group is located in the crevice between these domains. As seen in Fig. 2, the proximal side of the enzyme in g-HRP undergoes more conformational changes in comparison to the distal side. It is clear that residues 175-260 from the proximal side rotate in clockwise direction (22.89 degrees with 0.92 Å shift along axis).

**Figure 3:**
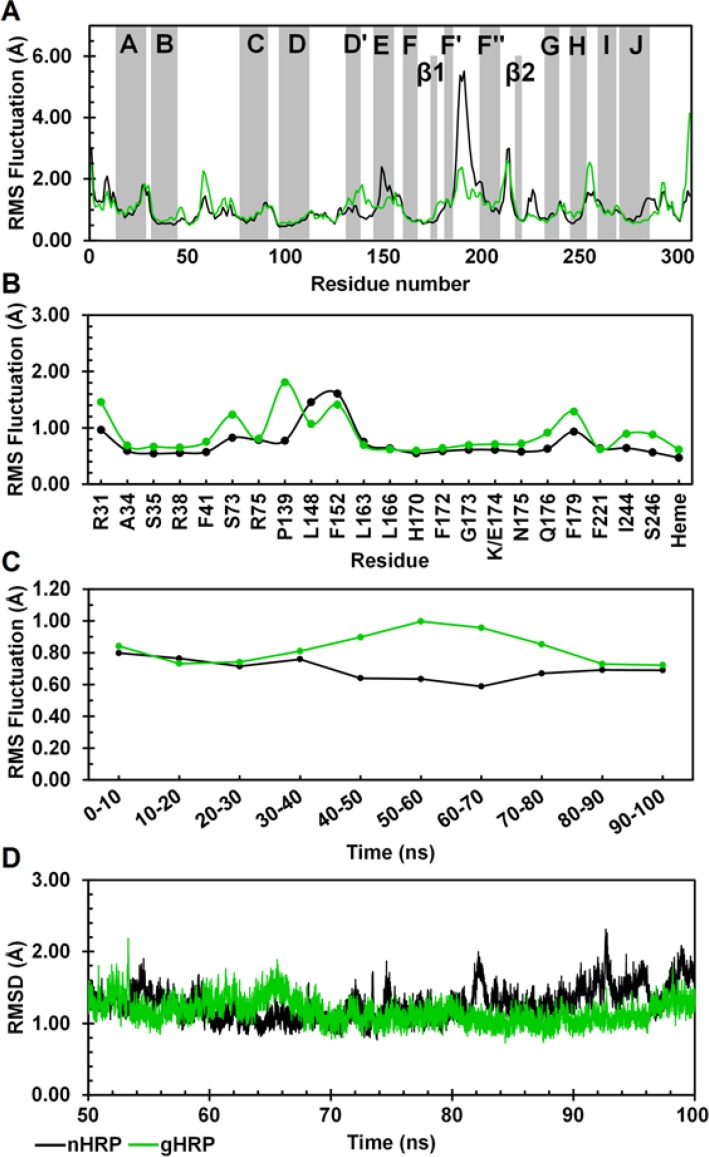
Per-residue RMS fluctuations for the whole protein backbone (A), and protein backbone around the heme as well as the heme prosthetic group (B) along the analyzed tim frames. The gray bands in the top panel indicate the helix and sheet regions of HRP according to the crystallographic structure. (C) Heme RMSF calculated for 10 ns time intervals during the entire course of 100 ns MD simulation. (D)Time dependence of the backbone RMSD with respect to the average structure during the analyzed time frames. n-HRP (black) and g-HRP (green) are reported.

The backbone root mean square fluctuation (RMSF) per residue for the native and modified HRP averaged over the analyzed time frames is shown in Fig. 3A. According to the crystallographic structure (PDB ID: 1ATJ), HRP contains thirteen α-helices and two short antiparallel β-strands. It seems that the mobility of regular secondary structures of g-HRP, except for the helices D' and E, is more or less similar to the native one. As shown in Fig. 3A, the helix E and also the connecting loop F'F" have experienced a decrease in mobility upon substitutions.

Despite the overall behavior of the protein backbone, the protein backbone around the heme prosthetic group displays increased mobility upon substitutions. Therefore, increased mobility of the heme is to be expected (Fig. 3B). To provide a more detailed description of the heme mobility as well as its neighboring residues, the RMS fluctuation of the heme was calculated for every 10 ns time intervals. As shown in Fig. 3C, within the first 40 ns of the simulation, RMS fluctuations of the heme in g-HRP are approximately similar to the native HRP. From 40 to 60 ns, the heme fluctuation increases and after reaching a maximum value at 50-60 ns, it begins to decrease. A similar trend was also observed in the case of protein backbone around the heme (data not shown). Fig. 3D shows the time evolution of the backbone RMSD as superimposed on the average structure of the analyzed time frames. As seen, g-HRP has approached to a less fluctuating structure after about 80 ns of simulation. Such a result is not unexpected since three mutations have been imposed to the protein structure and it takes a time for the mutant protein to find its right conformation.

**Figure 4:**
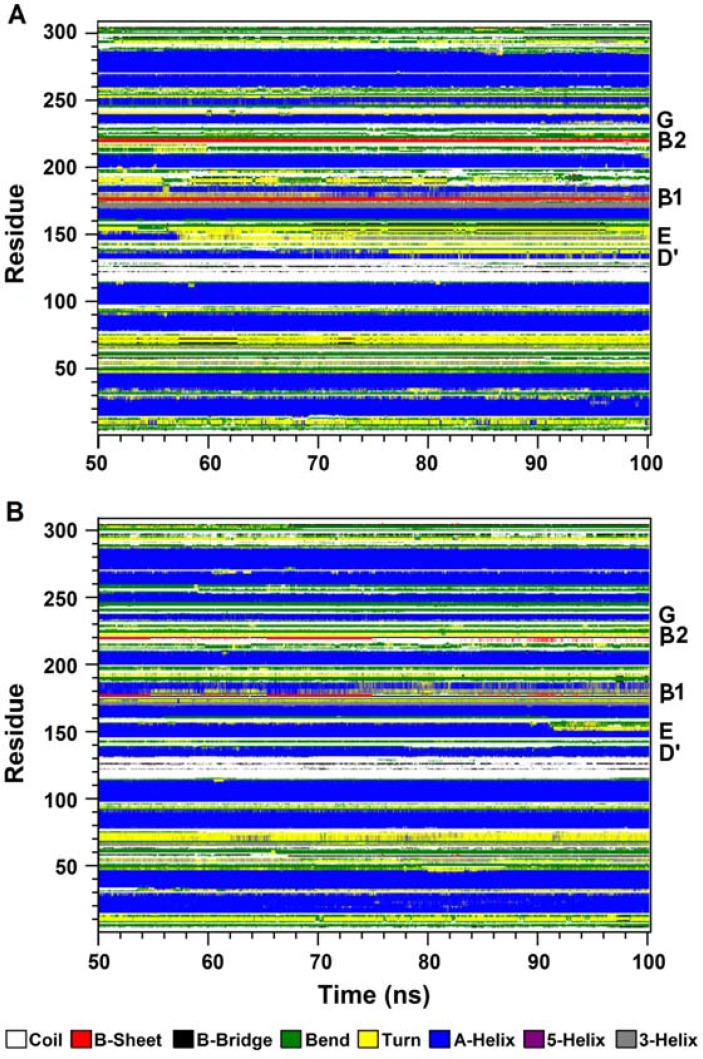
Time-dependent secondary structure fluctuations of n-HRP (A) and g-HRP (B) structures calculated using the DSSP program. The occurrence of secondary structure elements is indicated by using a color code

Analysis of the time-dependent secondary structure fluctuations using the DSSP program [36] provided a more detailed description of the conformational dynamics during the simulations (Fig. 4 and Table 1). Although in both models, the α-helices are dominant (Fig 4), values in Table 1 imply the α-helical content of HRP has increased after substitutions. Enhanced stability of the helices D' and E in g-HRP could be one of the reasons. In addition, DSSP analysis also revealed that several helices of g-HRP are longer and more stable than their counterparts in native HRP (Fig. 4).

**Table 1:** Occurrence of secondary structure elements calculated for the analyzed time frames of n-HRP and g-HRP simulations

**Secondary structure element**	**n-HRP**	**g-HRP**
**Coil,%**	23.20	23.91
**β** **-Sheet,%**	1.95	0.70
**β** ** -Bridge,%**	1.78	2.00
**Bend,%**	17.20	17.24
**Turn,%**	11.47	9.72
**α** **-Helix,%**	40.92	43.97
**5-Helix,%**	0.00	0.00
**3-Helix,%**	3.48	2.47

Analysis of the protein hydrogen bonds shows that the number of stable hydrogen bonds has increased in g-HRP. The total number of intramolecular hydrogen bonds with occupancy greater than 80% has increased from 98 in n-HRP to 103 in g-HRP (Table 2). In agreement, the existence percentage of hydrogen bonds forming helices D' and E in g-HRP is significantly higher than that of n-HRP (Table 3A-B).

To evaluate the effect of substitutions on the thermodynamic stability of HRP, protein stability changes (∆∆G) induced by substitutions were calculated using the Eris server [35]. The average structure of the n-HRP was submitted to the server and the prediction method was set to flexible backbone by allowing pre-relaxation. The residue positions, as well as the introduced residue types were identified for four prediction requests; K174E/K232E/K241E, K174E, K232E and K241E. The calculated ΔΔG values (−2.15, −1.40, −5.19 and −2.70 kcal mol-1, respectively) were all negative and implied that all individual and simultaneous substitutions have stabilizing effect on the protein structure, which were in agreement with the simulation results of g-HRP.

**Figure 5:**
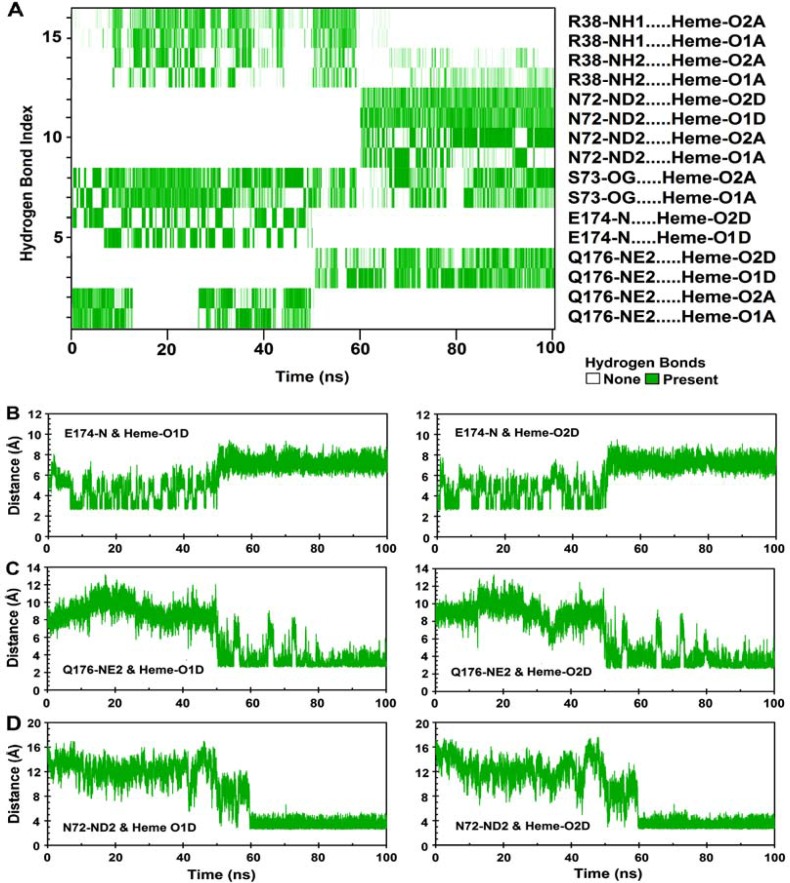
A) Heme-protein hydrogen bond existence map for g-HRP. Green lines show the presence of a hydrogen bond at that specific time. Time evolution of the pairwise distance for (B) Glu174-heme, (C) Gln176-heme, and (D) Asn72-heme interactions during the entire course of 100 ns MD simulation

**Figure 6:**
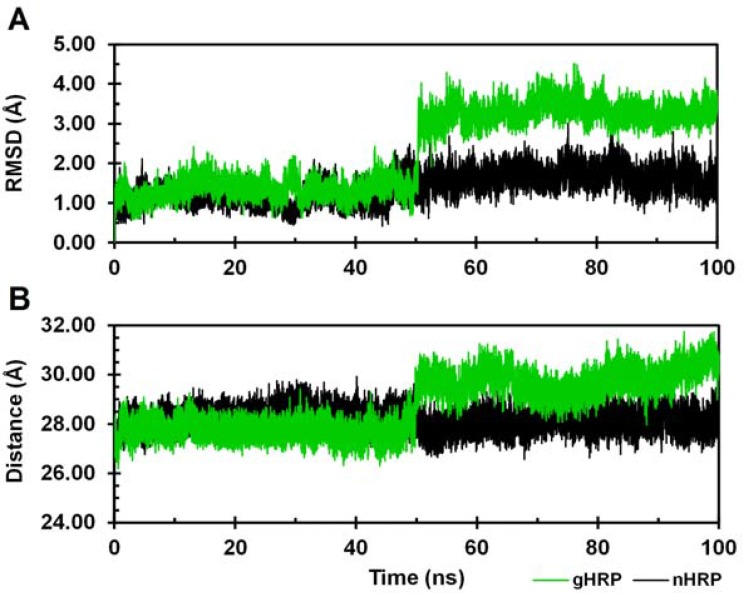
(A) Time dependence of the heme RMSD with respect to the starting structure. (B) Distance distribution between two Ca2+ ions during the entire course of 100 ns MD simulations. n-HRP (black) and g-HRP (green) are reported

To get a better insight into the consequences of these substitutions, local structural changes around the modification sites were investigated. As mentioned above, HRP contains a β-sheet comprised of two short antiparallel β-strands, β1 and β2. Three hydrogen bonds contribute in establishing this sheet (Table 3C). Residue 174 is located in strand β1, at the proximal side of the heme cavity. DSSP analysis of the n-HRP trajectories shows a persistent β-sheet structure throughout the simulation, while it is absent in 53.48% of the g-HRP analyzed trajectories (Fig. 4). In g-HRP, the β-sheet structure survives during the first half of the simulation, but after about 50 ns, the β- sheet becomes shorter and unstable and then is almost lost after ~75 ns. This is in accordance with the observed decrease in existence percentage of the hydrogen bonds involved in β-sheet formation in g-HRP model (Table 3C). Because of its proximity to the heme crevice, substitution of Lys174 could affect the hydrogen bonding network between the protein moiety and the heme prosthetic group. The backbone amide of Lys174 forms a hydrogen bond with the heme propionate oxygens. The hydrogen bond partner of the backbone amide of Lys174 is alternately switched between O1D (60.28%) and O2D (38.26%) of the heme, whereas in g-HRP they are absent in the analyzed time window. Further analysis revealed that during the first half of the g-HRP simulation, the heme prosthetic group is hydrogen bonded to Gln176, Glu174, Ser73, and Arg38 (Fig. 5A). Although these hydrogen bonds seem not to be very stable individually, their additive contribution can produce a relatively overall tight binding and hold the heme in its position. After about 50 ns, the hydrogen bonds between the nitrogen amide of Glu174 and heme propionate oxygens O1D and O2D (Figs 5A and B) are broken, probably due to the destabilization of the β-sheet conformation. This leads to clockwise rotation of the heme plane towards the loop D'E (Fig. 2). The obvious shift in the heme RMSD at ∼50 ns in Fig. 6A corresponds to breakage of these hydrogen bonds. The heme rotation causes the propionate oxygens O1D and O2D to be located within the hydrogen bond distance from NE2 of Gln176 (Figs 5A and C). This new position of the heme is further stabilized by the hydrogen bonds between ND2 of Asn72 and heme propionates, one of which alternately switches its partner between O1A and O2A (Figs 5A and D). In agreement, the RMSF value for the heme in Fig. 3C shows a steep decrease after 60 ns of simulation. This is in agreement with our previous proposition that the g-HRP has reached to its own structure after about 80 ns.

**Table 2:** Existence probability of hydrogen bonds during the analyzed time frames

**Existence percentage**	**n-HRP**	**g-HRP**
**90%≤**	63	66
**80%≤**	98	103
**70%≤**	121	124
**60%≤**	148	151
**50%≤**	171	180
**40%≤**	203	219
**30%≤**	254	255
**20%≤**	304	315
**10%≤**	415	434
**1%≤**	925	938
**Total**	2296	2372

Another interesting feature is observed in the distance between two structural calcium ions as the average value increases from 28.10 ± 0.41 Å in n-HRP to 29.80 ± Å in g-HRP. Detailed analysis of the distances between these Ca2+ ions throughout the simulation of g-HRP indicates that it has increased after about 50 ns (Fig. 6B) which is coincide with the onset of β-sheet structure instability. The proximal calcium binding site is located in a loop between the strand β2 and the helix G. Thr171 of the connecting loop Fβ1, which flanks the β1 strand, occupies one of the coordination sites of the proximal calcium ion. Hence, instability of the β-sheet makes this region more flexible in g-HRP and consequently causes Thr171OG1-Asp230O and Thr171N-Gly168O hydrogen bonds to be broken and attenuated, respectively. These hydrogen bonds have been compensated by the formation or improvement of other hydrogen bonds in vicinity of the second modification site. As a result, the loop β2G and its coordinated Ca2+ ion are pulled toward the helix F that makes the distance between two Ca2+ ions to be longer than the corresponding distance in n-HRP.

**Table 3:** Existence probability of hydrogen bonds involved in the formation of β-sheet and helices D', E and G during the analyzed time frames

Hydrogen bond index		Existence percentage, %	
		n-HRP	g-HRP
A) Helix D forming hydrogen bonds			
Ala134N	Ala134N	94.73	91.03
Asn135N	Leu131O	73.05	95.38
Ala136N	Asp132O	23.31	82.58
Asn137N	Leu133O	38.14	82.87
Leu138N	Ala134O	28.52	68.00
B) Helix E forming hydrogen bonds			
Leu148N	Leu148N	94.73	91.11
Lys149N	Lys149N	73.05	77.93
Asp150N	Asp150N	23.31	79.25
Ser151N	Ser151N	38.14	77.82
Phe152N	Phe152N	28.52	76.18
C) β-Sheet forming hydrogen bonds			
Asn175N	Leu148N	94.73	82.91
Cys177N	Lys149N	73.05	27.69
Val219N	Asp150N	23.31	96.74
D) Helix G forming hydrogen bonds			
Asn236N	Leu148N	91.11	89.57
Leu237N	Lys149N	77.93	91.31
Glu238N	Asp150N	79.25	-
Glu239N	Ser151N	77.82	-

Residues 232 and 241 are located in helix G and the connecting loop GH, respectively. Replacing Lys232 with Glu, strengthens the hydrogen bond between the backbone of residue 232 and Asn236N, and also causes the helix G to be shorter in length by two residues compared with n-HRP (Fig. 4 and Table 3D). This modification also strengthens the hydrogen bond between residue 232 side chain and OG1 atom of Thr225. The hydrogen bond acceptor is alternately switched between the OE1 and OE2 atoms of Glu232. Simultaneously, a new hydrogen bond between Thr225 and Asp220 is also formed in g-HRP. It is noteworthy to mention that residue 225 is involved in proximal Ca2+ binding pocket. Hence, this pocket is affected by the both substitution sites 174 and 232. In the third modification site, formation of some new hydrogen bonds together with a salt bridge, stitches the end of loop F'F" to the beginning of the connecting loop GH and enhance the integrity of this part of g-HRP structure. Glu241 and Gln240 from the connecting loop GH form new hydrogen bonds with Asn198 of the connecting loop F'F" in g-HRP. A salt bridge, which includes a built-in hydrogen bond, is formed between the side chains of Arg183 and Asp194. As a result, the mobility of the loop F'F" shows a clear decrease upon substitutions. This is evidenced by the observed reduction in the RMS fluctuation of the loop F'F" (Fig. 3A).

HRP catalyzes the oxidative coupling of phenolic compounds using hydrogen peroxide or organic peroxides as the oxidizing agent. The reaction is a three-step cyclic reaction in which the enzyme is first oxidized by H2O2 and then reduced in two subsequent one-electron transfer steps by reducing substrates, typically a small- molecule phenol derivative. The initial step in catalytic cycle of HRP involves the oxidation of the native enzyme to a short-lived catalytic intermediate, called compound I, by hydrogen peroxide [37]. This step has two distinct consequences; the reduction of hydrogen peroxide to water and the oxidation of the resting enzyme by two electrons. The Compound I intermediate contains an oxyferryl (Fe(IV)=O) center and an organic cation radical is located on the heme. To react with the heme prosthetic group, H2O2 has to diffuse from the protein surface toward the heme pocket. It is proposed that hydrogen peroxide penetrates the protein matrix at a fluctuating entry point located between Phe68 and Phe142 and passes through a bottle-like channel to reach the heme iron. Amino acid residues Phe68 and Phe142 are flanking the entry pore of the bottleneck, and their conformational fluctuations determine the accessibility of hydrogen peroxide to the interior [38]. Upon substitutions, the average distance between the backbones of Phe68 and Phe142 has increased from 10.34 ± 0.61 Å in n-HRP to 11.78 ± 1.49 Å in g- HRP. In agreement, the average distance between their side chains rises from 10.24 ± 1.15 Å to 12.23 ± 2.14 Å. Comparison of the RMS fluctuation of these residues also demonstrates significant increases in g-HRP. The backbone RMSF of Phe68 and Phe142 has increased from 1.09 and 0.76 Å in n-HRP to 1.34 and 1.34 Å in g-HRP, respectively. A similar increase was also observed for side chain RMSFs (2.37 to 2.83 Å for Phe68 and 1.33 to 2.33 Å for Phe142). These values suggest that the bottleneck entry has become wider and more flexible upon substitutions.

In the reaction of hydrogen peroxide with the active site of HRP, two residues in the distal heme pocket, Arg38 and His42, have been implicated in acid-base catalysis and cleavage of the peroxide O–O bond during compound I formation. It has been proposed that the distal His42 facilitates formation of the initial iron-peroxide complex by deprotonating the peroxide and subsequently, promotes cleavage of the oxygen-oxygen bond by protonating the distal oxygen [39]. Examination of the two models shows that the surface accessibility of this histidine in g-HRP (15.13 ± 8.0 Å2) is significantly higher than n-HRP (8.04 ± 5.49 Å2). This change could lead to enhanced reactivity of the modified enzyme to peroxide substrate.

**Table 4:** Solvent accessible surface area (Å2) of functionally important residues and groups averaged over the analyzed time frames

	**n-HRP**		**g-HRP**		
**Residue**	**Average**	**SD**	**Average**	**SD**	**Change**
**Phe68**	141.52	22.26	152.88	24.70	11.36
**Gly69**	9.67	5.77	18.00	8.25	8.34
**Leu138**	12.32	5.91	38.56	25.94	26.24
**Pro139**	10.09	5.59	32.47	15.52	22.38
**Ala140**	9.60	6.83	38.34	14.40	28.74
**Pro141**	22.14	10.30	46.52	15.54	24.38
**Phe142**	110.71	14.50	94.03	33.46	-16.68
**Phe179**	49.56	13.79	80.51	20.34	30.95
**Heme**	66.82	14.76	102.86	32.81	36.03

In the second step of HRP catalytic cycle, compound I oxidizes one aromatic reducing substrate molecule to give a substrate radical and compound II, where the organic cation radical is reduced to its resting state. Finally, compound II is reduced to the resting Fe(III) state of the enzyme by a second reducing substrate molecule [37] which is the rate limiting step in the catalytic cycle of HRP [40]. Aromatic substrates interact with HRP at the exposed heme edge, a region comprising heme methyl C18 and heme meso C20 protons. The substrate access channel starts with three peripheral Phe residues (Phe68, Phe142 and Phe179) and ends to the exposed heme edge. In addition to these residues, other hydrophobic residues such as Gly69, Leu138, Pro139, Ala140 and Pro141 surround the substrate access channel [27,41]. These residues together with the heme C20 and C18-methyl groups form the aromatic-binding pocket of HRP [27]. Most HRP substrates do not have the ability to make hydrogen bonds with the heme active site residues and will therefore depend more on the hydrophobic interactions with the peripheral regions of the substrate channel [42]. Accordingly, size of the reducing substrate, volume of the active site cavity, and extent of the hydrophobic patch are the major factors that could affect the reaction rate. The loop D'E (residues 138-144) covers one side of the inner wall of substrate access channel. Upon substitutions, longer and more stable helices D' and E (Table 3 and Fig. 4) cause the loop D'E to move toward the outside of the heme cavity, making the substrate access channel and its opening wider in g-HRP. On the other hand, values in Table 4 indicate that hydrophobic residues forming the substrate-binding pocket in HRP are totally more exposed in g-HRP, indicating that the surface of hydrophobic patch functioning as a binding site or trap for reducing aromatic substrates is extended in the modified enzyme.

Also, the results indicate that the accessibility of the exposed heme edge especially C20 and C18-methyl has been significantly increased after mutations (Table 4) mainly due to the rotation of the heme. Summing up the above simulation results, the conclusion is reached that such conformational changes at the aromatic substrate- binding site of HRP may lead to increased affinity and reactivity of the enzyme for aromatic substrates. The effects are expected to be less sensible for small substrates and significant for bulky ones.

## DISCUSSION

The stability of HRP can be considered in two respects; stability of the protein moiety and stability of the heme-protein interactions. Any modification could result in several small but subtle structural changes, spatially dispersed heterogeneously throughout the protein structure, some of which may improve the stability and others could act in opposite direction. The cumulative contribution of these changes will determine the final balance. It has been known that most of the stabilizing modifications of HRP target the solvent-exposed lysines 174, 232 and 241 [43]. In this study, the lysine residues 174, 232 and 241 were replaced by glutamic acid to investigate the effect of charge reversal at these positions on the structural properties of the enzyme. Stability increase has been reported upon experimental modification of HRP by negatively-charged anhydrides of phthalic, trimellitic, citraconic and maleic; of which phthalic anhydride also resulted in a catalytically more efficient enzyme [11-13, 44, 45]. Although, to our knowledge, there is no report on the simultaneous substitution of these three lysines by glutamic acid residues, it has been shown experimentally that simultaneous chemical modification of these lysines with charge neutralizing or reversing compounds leads to increased thermostability of HRP, while modification of all six lysines of HRP results in decreased stability [7]. Individual modification or substitution of lysines at positions 232 and 241 do not oppose with increased stability, but in case of Lys174, reported experimental as well as our simulation results tell a slightly different story.

In two separate experimental studies, both reported by Ryan et al., surface-exposed lysines of HRP were individually mutated to glutamic acid [14, 46, 47]. In first study, they analyzed the stability of 22 HRP mutants against hydrogen peroxide. The results showed that K232E single point mutation enhances hydrogen peroxide tolerance about 2-fold [46]. In the next study, they analyzed the effects of 13 single and three double point mutations on the stability of HRP. They reported that the single mutation K174E led to decreased thermal and solvent stability of HRP. Since all three single mutations K174A, K174E and K174N displayed decreased stability, they concluded that Lys174 replacement reduces the enzyme stability due to disrupting the hydrogen bond between Lys174 and the propionate oxygen of the heme prosthetic group [14]. Despite to Lys174, all of Lys232 mutants were found to be thermostabilizing with the exception of K232A. Since K241E mutation displayed a slightly lower T50 than wild type, they concluded that this mutation would not increase the thermostability of the enzyme [47]. However, the number of substitutions in these experimental studies and our simulations are not the same, so differences between their outcomes are not unexpected. This probability should not be ignored that upon simultaneous modification of all three lysine residues, each modification may modulate the effects of others.

In our simulations, analysis of the internal protein hydrogen bonds demonstrates that the number of stable hydrogen bonds (occupancy greater than 80%) and the α-helical content have increased in g-HRP. Although the helices D' and E are more persistent due to more and stronger hydrogen bonds in the modified enzyme, breaking of two hydrogen bonds in the C-terminal of helix G (including residue 232) causes the length of this helix to decrease. On the other hand, one of the β-sheet-forming hydrogen bonds has been relatively unstable after about 50 ns, thus this β-sheet, which flanks the substitution site 174, is absent in approximately half of the g-HRP trajectories. The majority of the structural changes observed in proximity of residue 174 are related to instability and consequent loss of this β-sheet.

In addition to protein-protein hydrogen bonds, these substitutions have also affected the hydrogen bonding network between heme prosthetic group and the surrounding residues. In native HRP, the heme moiety forms several hydrogen bonds with Arg31, Ser35, Ser73, Lys174 and Gln176 via propionate oxygens. Site-directed mutagenesis of HRP revealed that all three single mutations K174A, K174E, and K174N lead to decreased stability [47]. Accordingly, the authors concluded that this residue contributes significantly to the thermal stability of HRP and its substitution leads to heme disassociation due to the disruption of the heme-protein hydrogen bonds. To explain the reported stabilized derivatives of HRP which include chemically modified Lys174, they assumed that stabilizing chemical modifications only change the properties of Lys174 while the residue itself remains [47]. Lys174 makes hydrogen bond via its backbone amide nitrogen atom to the propionate oxygens of the heme. Since the backbone does not change upon the residue substitution, finding some Lys174 substitutions or modifications having no significant effect on the heme-protein hydrogen bonding is not far from expectation. In agreement, it has been reported that K174F replacement improves the heme-protein hydrogen bonding network [26]. Moreover, simultaneous substitution of all three surface exposed lysines can modulate the effects of each individual substitution on the protein structure. In this study, the hydrogen bond between Glu174 and heme propionate was broken after ~50 ns of simulation. It seems that β-sheet instability in g-HRP induces the breakage of this hydrogen bond and causes Thr171, one of coordinating residues of the proximal Ca2+, to move. As a consequence, heme rotates and so, the heme as well as the protein backbone around it experiences an increase in mobility. The new position of the heme in g-HRP is stabilized through a new hydrogen bonding network. This is supported by the observed decrease in mobility of the heme after about 60 ns of simulation.

These substitutions also increase the RMSF value of the heme and the backbone around it. In experimentally stabilized derivatives of HRP, it has been observed that thermostability of the modified enzymes increases due to the decreased conformational mobility of the protein backbone around the heme [7,16]. Here, at first glance, it may seem that the increased mobility of the heme in g-HRP results in decreased stability of the enzyme, whereas the RMSF analysis indicates that the observed increase in fluctuation is temporary and starts at 50 ns, exactly once the Glu174-heme hydrogen bond disrupts and begins to decline after 60 ns, as the new hydrogen bonding network between heme and protein moiety is established. Simultaneously with the reduction in heme RMSF, the backbone mobility of the heme neighboring residues has also been decreased.

In addition to the effects of the K174E substitution on the heme active site, the proximal calcium binding site is also affected by this substitution. Upon instability of the β-sheet, the loop β2G and its coordinated Ca2+ are pulled toward the helix F, a movement which raises the spatial distance between two Ca2+ ions in g-HRP. The resulting structural rearrangements are important since the substrate binding cleft is located between the two calcium ions. The next substitution site, Lys232, is located in helix G which in turn flanks the proximal Ca2+ binding pocket. The K232E replacement has strengthen the hydrogen bonds of residue 232 with Thr225 and Asn236; of which Thr225 is involved directly in coordination of the proximal Ca2+. Asn236 is also involved in the second coordination sphere of the proximal calcium ion through Asp222. Such changes together with formation of a new hydrogen bond between Thr225 and Asp220 could contribute to the stability of proximal Ca2+ binding pocket which is assumed to be essential for the structural and functional integrity of the enzyme [14,48]. The third substitution site, Lys241, is located in the loop GH. From the results obtained, it seems that improvement of the hydrogen bonding network together with formation of a new salt bridge between Arg183 and Asp194, enhances the stability of this region in g-HRP. Summing up the above mentioned results, it may be concluded that both the protein structure and heme-protein interactions are more stable in g-HRP, however, this stability has been achieved after 80 ns of the simulation. In fact, the mutated protein needed more time to adjust its structure with the imposed substitutions.

Mutagenesis and chemical modification of amino acids can also influence the catalytic properties. Since HRP has a very high catalytic turnover, some loss in its activity does not significantly affect its practical usefulness. In most reports, the activity of the stabilized forms of HRP has been described to be similar-to-wild type or with minor decrease. However, simultaneous increase in the stability and activity has also been reported in the enzyme modified by phthalic anhydride, glucosamine hydrochloride and anthraquinone 2-carboxylic acid [11,12,16,44].

Molecular dynamics simulations showed that substitutions improve the accessibility of His42 and the heme prosthetic group to the peroxide and aromatic substrates, respectively. Moreover in g-HRP, the bottleneck entry of the peroxide substrate access channel is wider and the hydrophobic patch functioning as a binding site or trap for reducing aromatic substrates is more extended. Such observations have also been previously reported for experimentally modified HRP with Anthraquinone 2-carboxilic acid [16]. Accordingly, higher affinity of the enzyme for its substrate is expected.
